# Prevention of Mother-To-Child Transmission of HIV: Cost-Effectiveness of Antiretroviral Regimens and Feeding Options in Rwanda

**DOI:** 10.1371/journal.pone.0054180

**Published:** 2013-02-20

**Authors:** Agnes Binagwaho, Elisabetta Pegurri, Peter C. Drobac, Placidie Mugwaneza, Sara N. Stulac, Claire M. Wagner, Corine Karema, Landry Tsague

**Affiliations:** 1 Ministry of Health, Kigali, Rwanda; 2 Division of Global Health Equity, Brigham and Women's Hospital, Boston, Massachusetts, United States of America; 3 UNAIDS-Ethiopia, Addis Ababa, Ethiopia; 4 Partners In Health – Inshuti Mu Buzima, Kigali, Rwanda; 5 Rwanda Biomedical Center, Kigali, Rwanda; 6 Partners In Health, Boston, Massachusetts, United States of America; 7 Global Health Delivery Partnership, Boston, Massachusetts, United States of America; 8 UNICEF-Zambia, Lusaka, Zambia; Africa Centre for Health and Population Studies – University of KwaZulu-Natal, South Africa

## Abstract

**Background:**

Rwanda's National PMTCT program aims to achieve elimination of new HIV infections in children by 2015. In November 2010, Rwanda adopted the WHO 2010 ARV guidelines for PMTCT recommending Option B (HAART) for all HIV-positive pregnant women extended throughout breastfeeding and discontinued (short course-HAART) only for those not eligible for life treatment. The current study aims to assess the cost-effectiveness of this policy choice.

**Methods:**

Based on a cohort of HIV-infected pregnant women in Rwanda, we modelled the cost-effectiveness of six regimens: dual ARV prophylaxis with either 12 months breastfeeding or replacement feeding; short course HAART (Sc-HAART) prophylaxis with either 6 months breastfeeding, 12 months breastfeeding, or 18 months breastfeeding; and Sc-HAART prophylaxis with replacement feeding. Direct costs were modelled based on all inputs in each scenario and related unit costs. Effectiveness was evaluated by measuring HIV-free survival at 18 months. Savings correspond to the lifetime costs of HIV treatment and care avoided as a result of all vertical HIV infections averted.

**Results:**

All PMTCT scenarios considered are cost saving compared to “no intervention.” Sc-HAART with 12 months breastfeeding or 6 months breastfeeding dominate all other scenarios. Sc-HAART with 12 months breastfeeding allows for more children to be alive and HIV-uninfected by 18 months than Sc-HAART with 6 months breastfeeding for an incremental cost per child alive and uninfected of 11,882 USD. This conclusion is sensitive to changes in the relative risk of mortality by 18 months for exposed HIV-uninfected children on replacement feeding from birth and those who were breastfed for only 6 months compared to those breastfeeding for 12 months or more.

**Conclusion:**

Our findings support the earlier decision by Rwanda to adopt WHO Option B and could inform alternatives for breastfeeding duration. Local contexts and existing care delivery models should be part of national policy decisions.

## Background

In 2009, an estimated 370,000 children were newly infected with human immunodeficiency virus (HIV) globally, primarily due to mother-to-child transmission (MTCT) [1]. Transmission can occur during pregnancy, labour, delivery, and breastfeeding. In the absence of interventions, the rate of vertical transmission is 20% or more [2]. In developed countries, the use of highly active antiretroviral therapy (HAART), elective caesarean section, and avoidance of breastfeeding has drastically reduced the risk of MTCT below 2%, yet these interventions are not always available in resources limited settings [3].

Compared to previously recommended regimens for the prevention of mother-to-child transmission (PMTCT) of HIV in resource-limited settings [4], recent studies in Botswana [5] and Malawi [6] have demonstrated greater effectiveness of various antiretroviral (ARV) regimens including maternal short course HAART (Sc-HAART) from 26–34 weeks of gestation through 6 months post-partum and extended infant nevirapine (NVP) prophylaxis for 28 weeks during breastfeeding in preventing MTCT including during breastfeeding. The World Health Organization (WHO) 2010 revised guidelines reflect this growing body of evidence [7]. For HIV-infected pregnant women not eligible for HAART, these guidelines offer 2 options during pregnancy and breastfeeding: (i) Option A (Dual maternal ARV prophylaxis plus extended infant ARV prophylaxis) – consisting of maternal daily zidovudine (AZT) from 14 weeks gestation through delivery, AZT+3TC during delivery and extended infant daily NVP from birth through 1 week after last exposure to breast milk and; (ii) Option B (maternal Sc-HAART prophylaxis) – consisting of daily maternal HAART from 14 weeks gestation until 1 week after cessation of breastfeeding, and 6 weeks of daily NVP or AZT for the infant. The recommended duration of breastfeeding by WHO for Options A and B is exclusive breastfeeding through 6 months and continued through 12 months with complementary food [7].

Against this context of evolving evidence and recommendations, countries are revising national PMTCT guidelines and are called to make informed policy choices based on local context, balancing the effectiveness, cost, feasibility and sustainability of various PMTCT regimens options. However, previous PMTCT cost-effectiveness studies have not included the range of PMTCT regimen options currently available and often did not adequately account for the cost of treating HIV-infected infants, given that many HIV-infected children now survive into adolescence and adulthood [8]–[13]. A recent economic analysis from Tanzania, which found that maternal antiretroviral therapy (ART) through pregnancy and breastfeeding was more cost-effective than single-dose nevirapine (sd-NVP), provides an updated economic analysis but is limited in its generalizability, given its context of 1 rural Tanzanian hospital [14]. To better inform countries' policy decisions, there is a clear need for more comprehensive cost-effectiveness analyses, which include current data on PMTCT options available in resource-limited settings.

In Rwanda, the prevalence of HIV among pregnant women is 4.3% [15]. An estimated 68% of HIV-positive pregnant women received some antiretroviral (prophylaxis or treatment) for PMTCT in 2009 [16]. Rwanda launched its national PMTCT program in 2001, using the sd-NVP regimen. In September 2005, national PMTCT guidelines were revised to reflect 2004 WHO recommendations for more effective multidrug (md)-ARV regimens. A successful transition from sd-NVP to multidrug-ART has been documented [17]. In November 2010, Rwanda adopted new national guidelines based on Option B based on the WHO 2010 recommendations, with the hypothesis that this option is more cost-effective than the other existing options. The current study aims to test this hypothesis through an analysis of the cost, effectiveness (children HIV infections averted and 18 months HIV-free survival) and savings of a range of PMTCT scenarios based on the Rwanda context. This will further assist policy makers in the analysis of the current course of action and provide useful insights to other countries' PMTCT programmes.

## Methodology

In 2010, authors calculated the cost and effects of different PMTCT scenarios for a cohort comprising the estimated 10,300 HIV-infected pregnant women needing PMTCT in Rwanda in 2009 [16] and adopted the perspective of the Government of Rwanda as a health care payer.

We compared PMTCT regimen options (Table 1) for HIV-positive pregnant women not eligible for lifelong HAART and HIV-exposed infants, as per 2010 WHO PMTCT guidelines [7] and 2010 Rwanda PMTCT guidelines [18]: Dual ARV prophylaxis and Short course HAART (Sc-HAART) prophylaxis extended throughout breastfeeding.

**Table 1:** PMTCT antiretroviral regimens options for HIV-positive pregnant women not eligible for lifelong HAART and HIV-exposed infant considered for the modeling, Rwanda 2010.

ARV regimen and infant feeding options	Mother ARV regimen	Infant ARV regimen
**Dual ARV prophylaxis and breastfeeding (WHO – Option A)**	Twice daily AZT from 14 weeks gestation or as soon thereafter as****possible; AZT+3TC during labour and delivery; 7 days AZT+3TC post-partum	Sd-NVP at birth, then once daily NVP for the first six weeks of life [49]
**Dual ARV prophylaxis and replacement feeding**	Twice daily AZT from 14 weeks gestation or as soon thereafter as****possible; AZT+3TC during labour and delivery; 7 days AZT+3TC post-partum	Sd-NVP at birth, then once daily NVP for the first six weeks of life [49]
**Short course (Sc)-HAART prophylaxis and 6, 12 or 18 months breastfeeding (WHO-Option B)**	Short-course (Sc) HAART prophylaxis from 14 weeks gestation****throughout the duration of breastfeeding period. First-line regimen****is TDF+3TC+NVP.	Sd-NVP at birth, then once daily NVP for 6 weeks
**Short course (Sc)-HAART prophylaxis and replacement feeding**	Short-course (Sc) HAART prophylaxis from 14 weeks gestation until****1 week post-partum. First-line regimen is TDF+3TC+NVP.	Sd-NVP at birth, then once daily NVP for 6 weeks

For Dual ARV prophylaxis, two infant feeding options were considered: 1) exclusive breastfeeding (BF) during 6 months and continuation of BF for the first 12 months (Option-A), and 2) replacement feeding from birth. For Sc-HAART, the infant feeding options were: 1) breastfeeding for 6, 12, or 18 months (Option-B), and 2) replacement feeding from birth. While 6 months of exclusive breastfeeding is currently recommended in Rwanda, recent data suggest that rate of exclusive breastfeeding in the general population is only 38% [19]. The average duration of breastfeeding in Rwanda in all women is 24.9 months [20].

The replacement feeding component was modeled on the Partners In Health (PIH) delivery model, operating in 2 rural districts in Rwanda since 2005. The model includes provision of 9 months of formula and related supplies, community health workers conducting home visits for direct observed ARV therapy, social worker home visits, regular support for formula preparation and active follow up of defaulters [21].

Under each scenario, all HIV-positive pregnant women eligible (CD4 count <500) for HAART treatment receive long course (Lc) HAART, initiated at time of diagnosis, for life. First-line regimen is tenofovir, lamivudine, and nevirapine (TDF+3TC+NVP). If CD4 is above 350, NVP is replaced by EFV after the first trimester. The infant ARV regimen includes Sd-NVP at birth, followed by once daily NVP for 6 weeks. In the absence of data for Rwanda, we considered 73% of HIV-infected pregnant women in PMTCT programs to be eligible for ART – as reported in the literature [22].

For the purposes of modeling and comparison across options, we included a scenario in which the mother-baby pair receive no intervention and assumed that in each scenario all women non-eligible for Lc-HAART were covered with the chosen option (while all eligible women received Lc-HAART under each scenario). We also assumed that the entire cohort of HIV-infected pregnant women discovers their HIV status when tested during antenatal care visits. Pregnant women who did not come for antenatal visits, who came late, who were identified during labor, or who had suboptimal regimens for any other reason such as low adherence were not considered in the base case model. Also, in Rwanda, where there is very high coverage of HIV counselling and testing services and of ART programs, some HIV-infected pregnant women are already aware of their HIV status when initiating PMTCT services and are enrolled in ART programs. Given that these women were eligible for treatment and were already receiving lifelong ART prior to ANC and PMTCT, we did not attribute additional ART costs to the PMTCT program for the purposes of the cost-effectiveness calculations (the daily NVP to baby that would be added to the mothers' existing regimen is only a small cost).

Table 2 provides details on how ARV regimens and feeding practices were distributed in the cohort of pregnant women for each study base case scenario.

**Table 2:** ARV regimen options and feeding practices distribution in each study scenario (for non eligible women) – base case.

Study scenarios	No intervention	Dual ARV breastfeeding	Dual ARV replacement feeding	Sc-HAART 6 mo. breastfeeding	Sc-HAART 12 mo. breastfeeding	Sc-HAART 18 mo. breastfeeding	Sc-HAART replacement feeding
*No prophylaxis,* **** *%*	100						
*Dual ARV,* **** *%*		100	100				
*Sc-HAART Prophylaxis,* **** *%*				100	100	100	100
*Mixed feeding first 6 months,* **** *%*	60 [19]	60	–	60	60	60	–
*Exclusive breastfeeding* **** *first 6 months,* **** *%*	38 [19]	38	–	38	38	38	–
*Mean duration of* **** *breastfeeding,* **** *months*	24.9 [20]	6 [7]	–	6	12	18	–
*Replacement feeding,* **** *%*	2 [50]	2	100	2	2	2	100

One-way sensitivity analysis was performed for the main inputs of the model: breastfeeding practices; cost of ART, laboratory tests, and replacement feeding; the proportion of eligible women; HIV transmission rates and HIV-free survival rates.

### Costs

Direct costs were modeled based on all inputs in each PMTCT option (including staff time and consumables) and related cost components. Since PMTCT in Rwanda is horizontally integrated with other services, we included only the extra cost of adding PMTCT services to existing activities and attributed a percentage of the indirect costs (capital and recurrent). We assume that if PMTCT services were to be established in new sites, infrastructure and other capital costs would be similar across PMTCT treatment options and, therefore, the ranking across options would not change. Using an exchange rate of 570 Rwandan Francs per USD [23], the list of inputs and data sources for each of the costed items is summarized in Table 3.

**Table 3:** Inputs, assumptions and data sources for costs of PMTCT interventions, Rwanda.

Items	Source
**ARV medications**	Protocols were taken from WHO 2010 guidelines adapted to the Rwanda context [7], [18] Prices of drugs were from Clinton Health Access Initiative (CHAI) price list, April 2010 [51] In addition to the price of drugs, were included: - Freight cost: 20% for syrups, 12% for pills - Central Purchasing of Essential Medicines in Rwanda (CAMERWA) Management costs: 9%
**Cotrimoxazole (CTX)**	2010 Protocols from the Center for Treatment and Research on AIDS, Malaria, Tuberculosis and Other Epidemics' (TRAC-Plus) ART guidelines [18] - CTX 25/5 mg/kg once daily for each child born to an HIV-infected mother in whom the diagnosis of HIV is not yet formally excluded - CTX 960 mg once daily for mothers during pregnancy and for 6, 12 or 18 months under Sc-HAART options CTX price was from CAMERWA, plus freight and management costs as per ARV medications
**Laboratory**	Quantities of exams varied according to PMTCT protocols. The following exams were included [7]: HIV Polymerase Chain Reaction (PCR) for early infant diagnosis (one to three times per child, 30 USD per exam); HIV rapid antibody tests (one to two times per child plus for pregnant women, 1.59 USD per test); CD4 cell counts (every 6 months per child, 20.7 USD per exam); hematology (two to four times per child, 4.9 USD each time); biochemistry (two to four times per child, 8.3 USD each time); Viral load (one time per child under Sc-HAART 12 and 18 months, 50.5 USD each time). Prices were provided by the 2007 quantification exercise from TRAC-Plus and CAMERWA and adjusted to 2009 USD equivalents [52]
**Staff time (personnel direct services)**	The following actions were costed: Administer or dispense ARVs; administer CTX; blood draw; charting on PMTCT; counselling about ARVs; counselling for infant feeding; family planning services; lab test for adult women and infants; offer or register for HIV testing; pre-test and post-test counselling (VCT); pre- and post-test counselling for patient and partner; administer ARVs. The frequency of each action per each of the scenarios was determined by consensus of the authors involved in PMTCT at the health centres. The cost of each action was taken as the average cost (type of staff, staff time and average salary cost including social security and taxes) in all Rwanda PMTCT facilities in 2009 as reported in the Paediatric HIV/AIDS Care Costing Study in Rwanda, 2010 Intermediate Report [53]. The referenced study recorded type of staff and staff time per each action by repeated observation in a representative sample of health centres in the country.
**Staff training**	The need for staff training was assumed as 3 to 5 days per PMTCT site, 4 staff trained for each site (consensus of authors). The expected useful life of training (rate of refresher training) was estimated at 2 years. The cost of staff training was as per the Global Fund to Fight AIDS, Tuberculosis and Malaria (GFATM) National Strategy Application budget, 67 USD per day per person all included [54]
**Promotion/mass media campaign**	The cost of a national promotion campaign: 190,000 USD was taken from the GFATM National Strategy Application budget [54]
**Contribution to the capital and running costs (overhead/administration) of a health center**	The financial analysis of 2 average health centers in Musanze District by CCHIPs (Comprehensive Community Health Initiatives & Programs) and CHAI in FY 2009 estimated 24,971 USD for logistics, utilities, material goods, maintenance and renovation, medical equipment and support staff. Since in an average health center in Rwanda, 5% to 10% of overall staff time is attributable to PMTCT activities (Rwanda MOH key informants): 2 days a week dedicated to PMTCT activities (2 full-time nurses out of 15 nurses, 10% of doctors' time), we attributed 10% of the overhead to PMTCT (the same amount across PMTCT options). We also added 10% of the annual amortization cost for a health center building [55]

For HIV-positive pregnant women (identified through PMTCT) eligible to ART, we calculated the cost of treatment for 18 months only, as this would contribute to 18 month HIV-free survival in breastfed children.

The replacement feeding component includes counseling for replacement feeding in the maternity ward after delivery (staff time of feeding assistants and midwives); follow-up home visits by community midwives with related staff time and transport costs (1 visit per week during first 2 months of enrolment and 1 visit per month during months 3–18); education at health centers by social workers (including time of staff, demonstration kits and transportation reimbursement for each participant); time spent by health staff in administrative issues and meetings related to milk distribution; material provided to mothers (small stove, thermos, jerry cans, baby bottles, pot); the cost of formula from birth until 9 months of age, using the least expensive bulk formula pricing available in Rwanda (6 tins per month for babies aged 0–2 months; 8 tins per month for babies aged 3–5 months; 4 tins per month for babies aged 6–8 months) and the cost of training staff (5 days on average per staff).

### Effectiveness

Two outcomes of interest were considered to evaluate PMTCT regimen effectiveness: the number of children HIV infections averted against “no intervention”, and 18-month HIV-free survival (number of children HIV uninfected and alive at 18 months). The transmission probabilities used to estimate the numbers of HIV infections in children born to HIV-positive mothers and the relative risks of mortality for HIV exposed uninfected children used to calculate 18-month HIV-free survival rates were extracted from the literature, including from two large reviews [24], [25], and are reported in Table 4. The widest ranges reported in the literature were used in sensitivity analysis to best account for uncertainty around the base case values.

**Table 4:** Transmission probability for each PMTCT protocol and feeding option and Mortality rates at 18 months (data refer to 12–24 months) for HIV exposed uninfected children used to calculate HIV-free survival.

Transmission probability percentage at birth (replacement feeding)	Base Case	Low range	High range
No intervention	20% [2]	13% [56]	30% [57]
Dual ARV (Option A, WHO)	4% [2]	2% [58], [59]	6% [58], [59]
Sc-HAART prophylaxis (Option B, WHO)	1.2% [60]	0% [60]	2.5% [60]

Breastfeeding transmission probability per month	Base Case	Low range	High range
1–6 months, exclusive breastfeeding	0.5% [2]	0% [61], [62]	3.3% [61], [62]
1–6 months, mixed breastfeeding	1% [2]	0% [61], [62]	6.6% [61], [62]
With Sc-HAART	0.33% (first 6 months mixed feeding)	0.08% [5]	0.4% [5]
	0.17% (first 6 months excl. breastfeeding and additional months) [5]		
With infant daily NVP during 6 months breastfeeding (Option A)	1.7% at 6 months [6]	0% [6], [61], [62]	2.9% [6]
7+ months – mixed feeding	0.75% [2]	0.65% [63]	0.85% [63]

Values used to calculate survival at 18 months for HIV exposed uninfected children	Base Case	Low range	High range
Breastfeeding for 12 months or more, mortality rate	5.3%, estimate at 18 months [64]	–	–
Estimate of Relative risk (RR) of mortality if breastfeeding for 6 months versus breastfeeding for 12 months or more	RR = 1.5*Assumption in the absence of published data (middle point)	RR = 1 [45], [46]	RR = 3 [24]**RR 3 refers to 24 months HIV-free survival if early weaning at 4 months
Estimate of RR if Formula Feeding versus breastfeeding for 12 months or more	RR = 2 [25]***Reported data from Pakistan and Senegal, with consideration that survival rates with national scale up may be worse than reported in the PIH program (used in low range)	RR = 1 [24], [36], [41], [47], [65]	RR = 6 [66] or higher [25]****Data from Ghana, as an example of RR higher than 6

### Savings

Savings correspond to the lifetime costs of HIV treatment and care (HAART, opportunistic infections, laboratory tests, care program) for the HIV infections averted among children or the average number of years of survival under treatment multiplied by the annual cost of treatment and care, adjusted for the rate of access to treatment and adherence. Costs were discounted using a 3% rate, as per common practice in cost-effectiveness analysis [26]. Costs of treatment for AIDS (acquired immune deficiency syndrome) include antiretroviral treatment (first- and second-line drugs), laboratory tests and home-based care. Research has shown that OIs are exceedingly rare among HIV-positive infants who had been immediately initiated on HAART [27]. The incidence of opportunistic infections (OI) is expected to be low given Rwanda's early adoption (in 2009) of immediate initiation of HAART for HIV-positive infants; thus, OI treatment costs were not included in inputs. Specific survival information for HIV-infected children in Rwanda is not currently available. However, studies on the impact of HAART on survival of children infected prenatally are available for some other developing countries [28], [29]. Based on the results from Banerjee *et al*., which reported the longest follow-up period, we assumed a survival rate of 15.1 years from the date of diagnosis [28]. We used a death rate of 6% and 6.9% at 1 and 2 years of HAART, respectively, as per a multicounty report in sub-Saharan Africa [29]. Assumptions and data sources for each of the costed items are summarized in Table 5. More detail and actual quantifications are available from the authors upon request.

**Table 5:** Inputs costed in the model and related sources for prices for treatment of HIV-infected children.

Items	Source
**ARV drugs**	Protocols are as per TRAC-Plus 2010 ART guidelines [18]. First-line: ABC+3TC+NVP, ABC+3TC+EFV, AZT+3TC+NVP, AZT+3TC+EFV (61% of children). Second-line: AZT+3TC+LPV/r, ABC+3TC+LPV/r, AZT+ABC+3TC+LPV/r (39% of children). Distribution of children less than 15 years old across the protocols (according to age/weight) was provided by the TRAC-Plus ARV quantification team, part of the CPDS (Coordinated Procurement and Distribution System). CHAI price list, April 2010 was used for prices of ARV medications [51]. CAMERWA management cost and freight costs were added to price of drugs
**Laboratory**	The following were considered: CD4 cell counts tests; biochemistry tests; hematology tests; viral load tests; other tests; consumables. Quantification and costs were provided by CPDS as per 2009 patient needs, prices and budget
**Care**	Care for children on ART (staff time) included: medical visits, social work consultations, monthly counseling groups, nutritionist time, home visits. Direct patient visits by doctors, nurses, social workers, CHWs are included. Other staff (clerks, etc.), are not included. No other costs (non-personnel operational costs such as vehicles, equipment, and infrastructure) were included. Costing reflects different ages: infancy (age 0–1) and young childhood (age 1–10), when more frequent doctor visits are required for dosage changes; and adolescents (age 11–15). Gross monthly salaries, according to Rwanda MOH base salary norms, were used. Current TRAC-Plus guidelines were used whenever possible. In other cases, PIH local experience in paediatric HIV care was used to estimate visit length, frequency, and staffing
**CTX**	Dosage of CTX as per Rwanda ART guidelines [18] and WHO 2006 [4]. Changes from Syrup to pills was taken at 6 kg or 7 months [18]. Prices for CTX were provided by CAMERWA, including freight and management costs

Children antiretroviral treatment (ART) costs apply to 90% of the cohort of HIV-infected children. This percentage was obtained assuming 95% access to ART (a realistic assumption for Rwanda given an estimated coverage rate of 77% in 2009 [16] and the ambitious targets for scale up [30]) and considering the current high level of adherence to treatment and low losses to follow-up [31]–[34]. The cost for the cohort of HIV-infected children was additionally adjusted for mortality rate as per Rwanda preliminary findings of DHS 2010 at different ages.

## Results

### Average cost

Table 6 outlines the average cost per HIV-infected mother and HIV exposed infant (mother-infant pair) under each PMTCT regimen. Dual ARV prophylaxis with breastfeeding (WHO-Option A) was the least expensive option at 738 USD per mother-infant pair, followed by Sc-HAART with 6 months breastfeeding (801 USD). The higher average cost per HIV-infected mother-infant pair is with Sc-HAART and 18 months breastfeeding (1,024 USD). Details of cost components are available from the authors upon request.

**Table 6:** Average cost per HIV-infected pregnant woman-infant pair – base case, USD.

Costing data	*Dual ARV* *breastfeeding*	*Dual ARV* *replacement* *feeding*	*Sc-HAART 6 mo.* *breastfeeding*	*Sc-HAART 12 mo.* *breastfeeding*	*Sc-HAART 18 mo.* *breastfeeding*	*Sc-HAART* *Replacement* *feeding*
**Prophylaxis**						
ART costs	420.96	409.84	485.09	530.28	575.46	439.90
CTX	15.75	5.80	18.66	23.20	28.16	5.80
Lab costs	165.57	138.63	160.88	247.38	281.81	138.03
Other (human cost)	4.27	3.86	4.97	5.89	6.78	4.29
Training	$28.14	$28.14	$28.14	$28.14	28.14	$28.14
PMTCT promotion	18.45	18.45	18.45	18.45	18.45	18.45
Capital and running costs	$84.85	$84.85	$84.85	$84.85	$84.85	$84.85
**Replacement Feeding**						
Counselling maternity ward after delivery		0.16				0.16
Home visits		4.74				4.74
Education at health center		8.64				8.64
Indirect costs staff		3.31				3.31
Material		29.12				29.12
Milk		242.53				242.53
Additional capacity (training)		3.35				3.35
**Total**	**$737.98**	**$981.41**	**$801.03**	**$938.19**	**$1,023.65**	**$1,011.31**

### Effectiveness

The findings from the analysis in Rwanda show that the fewest new HIV infections in children occur when HIV positive pregnant women receive Sc-HAART coupled with replacement feeding for infants (124 versus 3,987 in the no intervention scenario). The highest number of new HIV infections in children takes place when mothers receive Dual ARV and breastfeed for 12 months (865 new infections).

Across all Sc-HAART options throughout the breastfeeding period, the shortest breastfeeding period (6 months) averted the most HIV infections (286 new infections versus 488 in the case of 18 months breastfeeding). Dual therapy with replacement feeding is associated with 76 fewer vertical HIV infections than Sc-HAART during 18 months of breastfeeding (412 versus 488), but remains inferior to Sc-HAART during 12 months breastfeeding (387 new infections). Infections through long-term breastfeeding overcome the effects of a more effective regime of ARV prophylaxis.

Analysis of HIV-free survival, within the assumptions of the base case, changes the ranking across options. The highest numbers of HIV-uninfected children still alive at 18 months are found with Sc-HAART with 12 months breastfeeding (9,387 children) followed by Sc-HAART with 18 months breastfeeding (9,292). The other Sc-HAART scenarios (Sc-HAART with 6 months breastfeeding and with replacement feeding) still rank higher than Option A.

### Net costs (Cost-Savings)

The annual cost of treatment and care per child, according to age range, is provided in Table 7. When looking at net costs (savings on future treatment and care from HIV infections averted after subtracting the cost of providing PMTCT services), all options appear cost-savings at different degrees. The highest net costs are reached with Sc-HAART with 6 months breastfeeding (−13,912,837 USD) followed by Sc-HAART and replacement feeding (−12,721,758 USD). The least net costs are reached with Sc-HAART and 18 months breastfeeding (−10,410,727 USD). Since all scenarios are cost-saving, the cost effectiveness ratios of net cost over infections averted are not reported.

**Table 7:** Cost of treatment and care components per child per year, USD.

Cost per year	*Children 0−1 year old*	*Children 1−10 years old*	*Children 11−15 years old*
**ART**	374.66	374.66	374.66
**Laboratory**	87.17	87.17	87.17
**Other care**	84.16	63.23	56.41
**CTX**	12.33	2.45	3.27
**Total**	**558.32**	**527.52**	**521.52**

**Figure 1:**
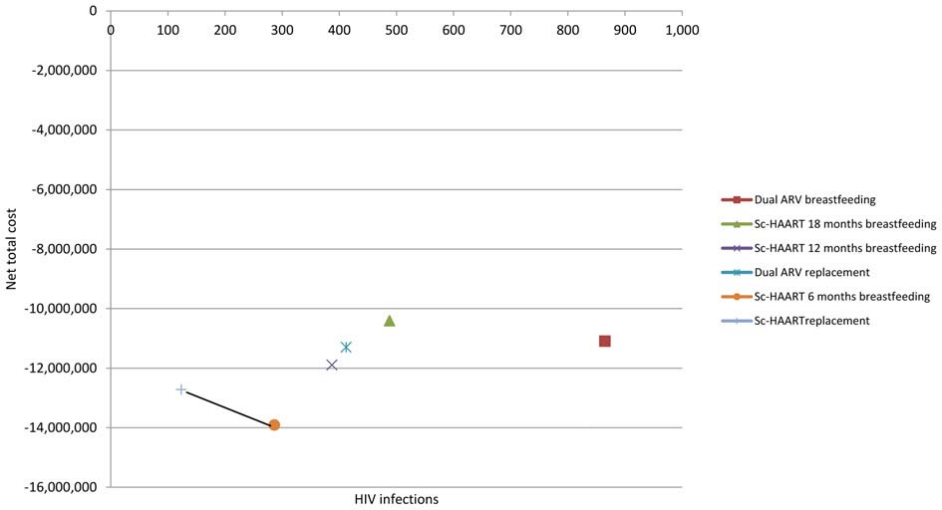
Number of new HIV infections occurring in children and net total cost, USD.

Figure 1 shows the number of HIV infections occurring in children together with the net total cost for each of the PMTCT scenarios.

**Figure 2:**
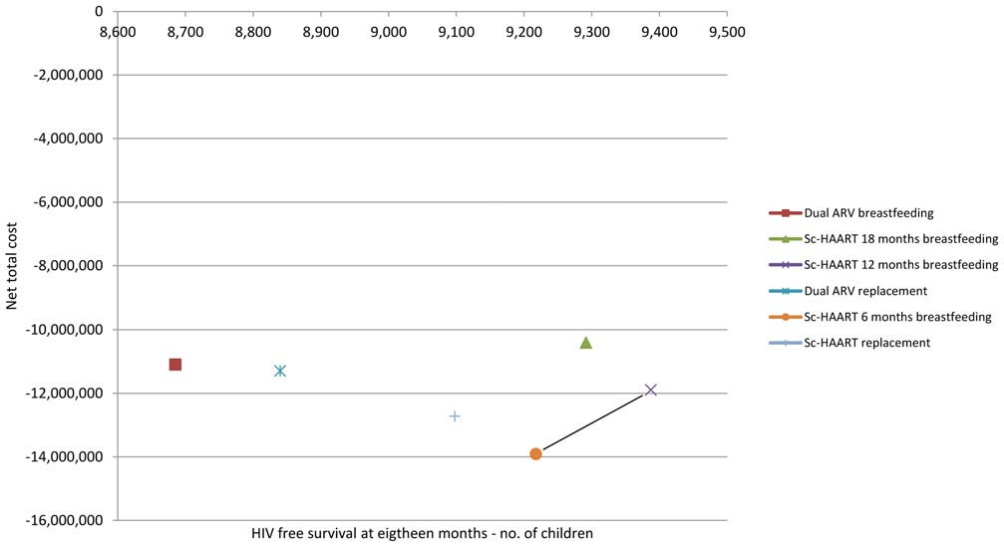
HIV-free survival (at 18 months) and net total cost, USD.

Figure 2 shows the number of children uninfected with HIV and alive at 18 months together with the net total cost for each of the PMTCT scenarios.

### Incremental cost – effectiveness analysis

When ranking scenarios based on the number of HIV infections, the analysis showed two alternatives averting more HIV infections and saving more money than all the other scenarios (Figure 1): Sc-HAART with 6 months breastfeeding and Sc-HAART with replacement feeding. Sc-HAART with replacement feeding averted 163 additional HIV infections than Sc-HAART with 6 months breastfeeding for an incremental cost of 1,191,079 USD and Incremental Cost Effectiveness Ratio (ICER) of 7,319 USD.

When ordering scenarios based on the number of children alive and uninfected, ICER analysis shows that there are only two alternatives that are not dominated (Figure 2): Sc-HAART with 6 months breastfeeding and Sc-HAART with 12 months breastfeeding. Sc-HAART with 12 months breastfeeding allows 170 additional children alive and HIV-uninfected at 18 months for an incremental cost of the program of 2,017,310 USD. ICER equals to 11,882 USD.

### Sensitivity Analysis

Given the uncertainty embedded in the input values of the base case scenario, we conducted a one-way sensitivity analyses for key variables of the model.

### Breastfeeding practices

To investigate the sensitivity of findings to breastfeeding practices, ranges were used for both breastfeeding duration and for the percentage of exclusive breastfeeding. As per recommendations, the base case uses 6 months as actual duration of breastfeeding (for options other than Sc-HAART for 12 and 18 months). However, anecdotal reports from health providers in Rwanda suggest that breastfeeding duration for HIV-infected women may be higher than the recommended 6 months due to lack of financial means. Therefore, we took 18 months as the low range (or worst scenario) in terms of actual breastfeeding duration in cases when health providers recommend 6 months to the mothers. For the percentage of women practicing exclusive breastfeeding during the first 6 months, the base case uses 38% as per the rate in the general population [19]. In reality, this percentage could be lower (we took a token 50% decrease from the base case, or 19%, in the absence of data) or higher. The proportion of mothers who practice exclusive breastfeeding may be higher since HIV-infected women are the more aware of the HIV transmission risk. A recent study from Uganda shows that up to 92% of women on ART exclusively breastfed their infants for a median duration of 4.0 months and stopped breastfeeding at a median age of 5.0 months [35].

Even in the most favorable of the breastfeeding scenarios (with 92% of mothers exclusively breastfeeding for the recommended 6 months), the highest number of HIV infections are averted by Sc-HAART with replacement feeding. The second best option in terms of number of infections averted remains Sc-HAART with 6 months breastfeeding. As expected, an extra infection averted through Sc-HAART with replacement feeding against Sc-HAART with 6 months' breastfeeding would cost more (an extra 14,229 USD versus 7,319 USD in the base case). In the worst case scenario for breastfeeding practices, when the percentage of mixed feeding and the actual breastfeeding duration increase to 18 months (independently of the duration of HAART), the number of HIV infections averted with the breastfeeding options (other than Sc-HAART for 18 months) decreases substantially. Therefore, Sc-HAART with replacement feeding becomes even more favorable and this scenario dominates all the others (the highest number of HIV infections averted for saving additional money).

When looking at HIV-free survival as an outcome of interest, we find that across the range of values for breastfeeding practices, Sc-HAART with 12 months breastfeeding still allows more HIV uninfected children alive at 18 months than Sc-HAART with 6 months breastfeeding and all other scenarios are dominated, The extra cost per child alive and uninfected is of 11,780 USD in the worst case scenario for breastfeeding practices and 10,774 USD in the most favorable breastfeeding scenario.

We conclude that our ranking across PMTCT scenarios is robust, or not sensitive, to breastfeeding practices.

### Costs of ARV, replacement feeding, and laboratory tests

Acknowledging that the cost of inputs may vary over time, we explored the variability of our results for potential 50% cost increases or decreases in the inputs that account for the highest proportion of cost, namely: ARV cost, laboratory costs and the cost of replacement feeding. As expected, if prices of ARV and laboratory tests increase, the replacement feeding options become more favourable. If prices of ARV and laboratory tests decrease, Sc-HAART scenarios become more cost-effective since they become cheaper. The opposite occurs when cost of replacement feeding increases. When ARV costs increase, savings also increase.

When looking at HIV infections averted, we found that even with varying input costs, Sc-HAART with replacement feeding and Sc-HAART with 6 months breastfeeding remain highest in the ranking across PMTCT options. When all costs increase by 50%, Sc-HAART with 6 months breastfeeding still dominates all scenarios, with the exception of Sc-HAART and replacement feeding, which allows more HIV infections averted for an extra cost of 11,643 USD per extra HIV infection averted. When all costs decrease by 50%, the same is true and the extra cost per extra HIV infection averted becomes as low as 2,995 USD.

When looking at HIV-free survival as an outcome of interest, Sc-HAART with 12 months breastfeeding and Sc-HAART with 6 months breastfeeding remain the dominant scenarios across the range of cost variations (+/−50%). The ICER for Sc-HAART with 12 months breastfeeding versus Sc-HAART with 6 months breastfeeding increases to 16,590 USD when costs increase and it decreases to 7,175 USD when costs decrease.

Our findings are therefore not sensitive to potential variations (estimated at a maximum of ±50%) in input costs.

### Proportion of women eligible for ART

With respect to the new eligibility criteria of a CD4 threshold of 500, data concerning the proportion of eligible women in Rwanda were not available. Thus we assumed that 73% of women in PMTCT programs may be eligible for ART, as reported in the literature [22]. Given the uncertainty of this value in the Rwanda context, we explored the effect on the results if only 36% of women were eligible to ART (CD4 less than 500). This is a 50% decrease comparing to the base case value.

With 36% of women eligible to ART, we found that when looking at HIV infections averted as an outcome of interest, Sc-HAART with 6 months breastfeeding still dominates all scenarios, with the exception of Sc-HAART and replacement feeding, which allows more HIV infections averted for an extra cost of 3,415 USD per extra HIV infection averted. When looking at HIV-free survival as an outcome of interest, Sc-HAART with 12 months breastfeeding and Sc-HAART with 6 months breastfeeding remain the dominant scenarios. Sc-HAART with 12 months breastfeeding allows more HIV uninfected children alive at 18 months for an increased extra cost of 15,639 USD per uninfected child alive at 18 months. Our results are thus not sensitive to plausible changes in the proportion of eligible women in the cohort of HIV-positive pregnant women.

### HIV transmission rates for each PMTCT option

Ranges for the HIV transmission rates, as per the most recent data available in the literature, are provided in Table 4. The highest and lowest values were used. Analysis for the lower bound is the more important considering that 500 CD4 was used as a cut off for ART eligibility in this study (as per Rwanda guidelines) and this may reduce the estimates of transmission as reported in the literature for a 350 CD4 cut off. As expected, there is a higher number of new HIV infections in situations with a higher HIV transmission probability. However, the overall ranking across scenarios does not change.

For HIV infections averted as an outcome of interest, when the low transmission rates are used, Sc-HAART with 6 months breastfeeding dominates all options with the exception of Sc-HAART with replacement feeding. However, the extra cost for an extra infection averted with Sc-HAART and replacement feeding versus Sc-HAART with 6 months breastfeeding increases to 38,712 USD. When the high transmission rates are used, the extra cost for an extra infection averted with Sc-HAART with replacement feeding versus Sc-HAART with 6 months breastfeeding decreases to 2,951 USD.

For HIV-free survival as an outcome of interest, Sc-HAART with 12 months breastfeeding and Sc-HAART with 6 months breastfeeding still dominate all other scenarios across the ranges of HIV transmission rates. The ICER for Sc-HAART with 12 months breastfeeding versus 6 months breastfeeding decreases to 7,542 USD when low HIV transmission rates are used and it increases to 94,544 USD when high transmission rates are used.

### Mortality rates at 18 months for HIV exposed uninfected children

In the absence of national data for Rwanda, the Relative Risks (RR) of mortality rates for children breastfeeding for only 6 months or being formula fed were drawn from the literature (see Table 4). However, several assumptions had to be made. For example, as no study was found on the RR for mortality for 6 months breastfeeding versus 12 months or more, a RR of 1.5 was taken as the middle point between the worst case of RR of 3 (when weaning at 4 months [24]) and the best scenario in which there would be no difference in survival outcomes. In the case of survival rates for replacement feeding, we chose a conservative approach for the best case. Rather than using a RR of 0.5 as reported in the PIH program [36], we assumed that survival rates for such a program may be less optimal in the context of a national scale up, and therefore selected a best case, low scenario, RR equal to 1.

**Figure 3:**
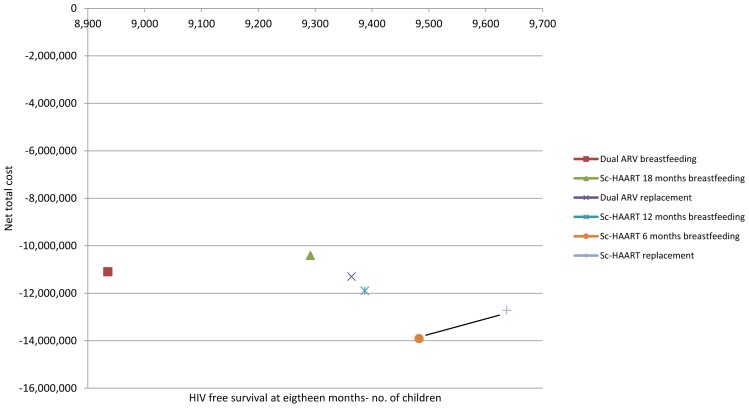
HIV-free survival (at 18 months) and net total cost, USD – sensitivity analysis – low range, RR = 1.

When redoing the analysis with this lower range (RR = 1), Sc-HAART with replacement feeding allows the highest number of children alive and uninfected at 18 months, for an extra cost per child of 7,745 USD compared to Sc-HAART with 6 months breastfeeding. All other options are dominated (see Figure 3).

When looking at the threshold at which the rankings of scenarios change, we found that Sc-HAART with replacement feeding becomes the scenario with highest HIV-free survival attainments if RR is 1.4 or lower. This would require a setting where replacement feeding with an available, nutritionally adequate and safe diet could be provided, such as in the model described in our study. For Sc-HAART with 6 months breastfeeding to become the favourite option in terms of HIV-free survival attainments, RR should be of 1.1 or below. This RR of mortality rate at 18 months might be the actual rate in some context, depending from the conditions of mothers/infants (and the support provided to them) during the weaning period and in the months that follow. When the analysis is re-done using the higher RR ranges (Table 4), Sc-HAART with 12 months breastfeeding allows the highest number of children alive and HIV uninfected at 18 months, for an extra cost per child of 2,089 USD compared to Sc-HAART with 6 months breastfeeding, dominating all other options.

We conclude that when using HIV-free survival as an outcome of interest, our results are sensitive to the assumptions used for the mortality rates among HIV exposed uninfected children, and therefore to the conditions (adequacy, safety) in which early weaning (after 6 months breastfeeding) or replacement feeding would take place.

## Discussion

This study modelled cost-effectiveness of various PMTCT ARV regimens and infant feeding options in the Rwandan context to inform policy dialogue at a time of increasing need for more value for money in global health interventions, and a global commitment to the virtual elimination of MTCT by 2015 [37], [38]. This is the first cost-effectiveness analysis of PMTCT regimen options that covers most of the regimen options available today in resource-limited settings, and uses HIV-free survival as the outcome of interest. Policy analysis of PMTCT regimens should prioritize child and maternal survival, therefore decision-making regarding solely HIV infections averted should be avoided.

This study shows that all PMTCT regimens under analysis are cost saving compared to “no intervention”. This indicates that investing in PMTCT services does bring future savings when taking into account the reduced needs for treatment and care services for children in whom infection is averted. As such, it is a moral and practical imperative for governments to aim for the highest number of children alive and HIV-uninfected (HIV-free survival) within an affordable PMTCT program.

Sc-HAART with 12 months breastfeeding and Sc-HAART with 6 months breastfeeding dominate all other scenarios when considering 18-months HIV-free survival. Sc-HAART with 12 months breastfeeding allows the highest number of children alive and HIV uninfected at 18 months, for an incremental cost per extra child of USD 11,882. However, this finding is very sensitive to the values used for the relative risk of mortality comparing children who are breastfed for 12 months or longer to exposed but HIV-uninfected children who are breastfed for 6 months or those who are formula fed from birth. Given the uncertainty embedded in these mortality assumptions, it is difficult to draw unidirectional conclusions. Cost-effectiveness findings for HIV-free survival are sensitive to the actual feasibility of providing nutritionally adequate and safe replacement feeding. WHO has recently issued programme guidance supporting the non-discontinuation of HAART after initiation among all HIV positive pregnant women (Option B+) [39]. Although our paper was not designed to specifically model this scenario, one could anticipate that the outcomes of interest in our study (children HIV infections averted and 18-months HIV-free survival) would not change significantly for Option B+ compared to Sc-HAART when measured at the end of the breastfeeding period. If the cost is higher under Option B+, this could be offset by the additional health benefits to women from earlier initiation of treatment and non-discontinuation. Other benefits would be reduced heterosexual transmission risk to male partners. We highly recommend further analysis on the cost-effectiveness of Option B+.

When considering HIV infections averted, the most cost-effective PMTCT regimens are Sc-HAART for 6 months post-partum with exclusive breastfeeding and Sc-HAART with replacement feeding. A recent analysis from Malawi also suggested that Sc-HAART with 6 months breastfeeding was cost effective for PMTCT [40]. Yet this study did not look at HIV-free survival [41]. Our sensitivity analysis showed that the ranking across PMTCT options is robust to plausible changes in values of key inputs parameters. Sc-HAART with replacement feeding prevents more infections than Sc-HAART with 6 months breastfeeding for an incremental cost of USD 7,319 per incremental HIV infection averted. When a mother can afford or be provided an adequate, safe, sustainable, and culturally acceptable substitute of breast milk, Sc-HAART with replacement feeding can be recommended. However, at the population level, the benefit of replacement feeding should be balanced with the increased child morbidity and mortality risks associated with abstaining from breastfeeding or early cessation of breastfeeding, particularly in resource-limited settings. There is evidence suggesting an increased risk of mortality, morbidity and slower early growth among HIV-exposed children than their HIV-unexposed counterparts [42]–[44]. Recent studies have nevertheless reported no significant increased risk of child mortality; however this is only possible when individualized community-based follow-up systems are implemented and direct support (milk, safe water, cooking equipment) is provided to mothers [36], [45]–[47].

An important limitation of our study is that the theoretical model excludes the possible impact of suboptimal treatment delivery resulting from poor adherence rates, losses to follow up, and suboptimal timing of ARV initiation. In addition, non-adherence to the different interventions considered would each have distinct impacts on our study's endpoints. Results are based on the ideal manifestation of the data, without taking into account possible deviations, for the main purpose of ranking options for decision making. Since fewer than 100% of women enroll and completely adhere to PMTCT [48], the real number of infections averted would be less than anticipated by our model.

In Rwanda, the national ART policy states that all persons living with HIV should have universal access to care and treatment for HIV. Therefore, the additional cost for each child who becomes infected is an increased – and lifelong – cost for the Government of Rwanda as the health care payer. Although our model uses the longest surviving paediatric cohort currently available in the literature [28], AIDS is a chronic illness and it is clear that care and treatment costs would continue to accumulate as children enter adulthood. As HIV care and treatment advances continue, improved survival in children with HIV would also be expected to increase. As such, the more efficacious PMTCT options would become increasingly cost-effective. Therefore, our model is somewhat conservative when estimating the potential savings from PMTCT options.

## Conclusion

Our analysis demonstrates that all PMTCT regimen options under analysis are cost saving relative to no PMTCT intervention. Failure to intervene to prevent MTCT is therefore unacceptable from an ethical, financial and public health perspective. Still, the quest for an HIV free generation should be considered within the broader maternal health, and child survival and development contexts.

Our findings support the earlier decision the Government of Rwanda made to adopt Option B (Sc-HAART) of the WHO 2010 ARV guidelines for PMTCT. Sc-HAART with 12 months breastfeeding bears the most benefit for HIV-free child survival from a public health perspective. Resource-constrained countries must consider their local context and health care delivery framework to weigh the risks and benefits of each option when setting national PMTCT policies.
